# Harvesting the maximum length of sciatic nerve from adult mice: a step-by-step approach

**DOI:** 10.1186/1756-0500-7-714

**Published:** 2014-10-10

**Authors:** Usman Bala, Kai-Leng Tan, King-Hwa Ling, Pike-See Cheah

**Affiliations:** Department of Human Anatomy, Faculty of Medicine and Health Sciences, Universiti Putra Malaysia, 43400 Serdang, Selangor Malaysia; NeuroBiology and Genetics Group, Genetics and Regenerative Medicine Research Centre (GRMRC), Faculty of Medicine and Health Sciences, Universiti Putra Malaysia, 43400 Serdang, Selangor Malaysia; Department of Human Anatomy, College of Medical Sciences, Gombe State University, P.M.B 127 Gombe, Nigeria; Clinical Genetics Unit, Department of Obstetrics and Gynaecology, Faculty of Medicine and Health Sciences, Universiti Putra Malaysia, 43400 Serdang, Selangor Malaysia

**Keywords:** Peripheral nerves, Sciatic nerve, Dissection, Maximum length, Biomedical research

## Abstract

**Background:**

Over the past several decades, many studies concerning peripheral nerve damage or regeneration have been performed. Mice have been widely used for many of these studies, with the sciatic nerve being the most targeted and preferred nerve. Therefore, techniques for harvesting mouse sciatic nerves of a maximum length that is sufficient for different analyses will be highly valuable. Here we describe a simple step-by-step guide for harvesting the maximum length of mouse sciatic nerve and compare the length of the harvested nerves gathered with the proposed method with nerves obtained using a conventional mid-thigh incision approach.

**Findings:**

The sciatic nerve was exposed while holding both hind limbs together in one hand and the tail was gently pulled away in the opposite direction. The nerve was traced by dissecting through its course both distally and proximally and was carefully harvested. The total average length of the sciatic nerves obtained using the proposed harvesting method and the mid-thigh incision method was 22.60 ± 1.62 mm and 7.0 ± 0.76 mm, respectively. This length of harvested nerve allows further dissection into several segments that can be used for additional independent analyses such as histochemical/histological analysis and RNA or protein extraction.

**Conclusion:**

The approach described here has several advantages over mid-thigh incision methods in that it: i) allows harvesting of maximum lengths of the sciatic nerve ii) allows simultaneous harvesting of both sciatic nerves, iii) provides time savings; iv) requires no extensive knowledge of veterinary anatomy; and v) provides hassle-free dissection.

## Background

Mice have been the most widely used animal model in biomedical and clinical research for several decades, largely because of their relatively short breeding circle, ease of maintenance and genetic similarities with humans [1]. Extrapolation of results obtained from animal models into human applications has been quite helpful in various fields of study, including drug development [2, 3], developmental toxicity [4], aging [5] and determining the role of developmental genes [6]. In terms of research on nerve injury therapies, peripheral nerves are the most targeted for studies concerning chronic constriction injury [7–9], partial sciatic nerve injury [10, 11] and nerve crush injury [12]. Analysis of affected nerves involves several protocols such as identification of the nerve region or exact localization of the injured nerve segment. The sciatic nerve has a similar fundamental anatomy in most animals. In rodents such as mice and rats, this nerve originates from spinal segment L3-L4 and L4-L5, respectively, with little contribution from L3 in rats [13, 14]. The neural segments that form the sciatic nerve in both mice and rats have functional homology [14].

In most neurological research involving studies on the mechanism of nerve regeneration [15, 16] or nerve degeneration [17], the sciatic nerve is the most preferred and widely chosen nerve. Similarly, this nerve is targeted in studies that involve assessing the animal for improvements in motor performance, reduced motor impairment and functional recovery after nerve injury or damage [18, 19]. The fact that the sciatic nerve is the largest nerve in the body and its course spans from the gluteal region to the popliteal fossa, makes this nerve the most frequently chosen for use in peripheral nerve-related studies.

Several studies demonstrated harvesting of the sciatic nerve from the lateral aspect of the animal’s thigh wherein only the segment of the nerve around the thigh region is harvested [8, 18]. However, in mice the larger portion of the nerve runs along the lumbosacral area, which is often not easily accessible. Our current research required sufficient amounts of mouse sciatic nerve tissue to allow subsequent independent downstream assays such as histochemical/histological analysis and RNA or protein extraction. This requirement motivated us to seek alternative approaches for harvesting the maximum length of the sciatic nerve. To the best of our knowledge, there are no publications describing such step-by-step approaches for harvesting maximal lengths of mouse sciatic nerves. We detail here a simple step-by-step dissection guide for harvesting maximal lengths of sciatic nerve in adult mice. We further compared the length of sciatic nerve obtained via the current dissection method with a conventional mid-thigh incision approach.

## Method

### Materials

Dissecting tools: forceps - curved blunt forceps with serrated tips and larger forceps with a curved blunt tip. Surgical scissors: sharp pointed and ligature scissors. Scalpel handle with a surgical blade (size-11). Dissecting board, 4% (v/v) isoflurane, 70% ethanol, pins, cotton buds and tissue paper.

### Dissecting protocol

#### Animal preparation and housing

- Ethical approval (Ethical no. UPM/FPSK/PADS/BR-UUH/00494) was obtained from the Animal Care and Use Committee (ACUC), Faculty of Medicine and Health Sciences, Universiti Putra Malaysia (UPM). Animal handling was performed in accordance with ACUC guidelines.
- A total of 12 adult male mice (C57BL/6), aged postnatal-day 70–84 and weighing between 24–28 g were obtained from the Mouse Room Facility, Medical Genetics Laboratory, Faculty of Medicine and Health Sciences, Universiti Putra Malaysia. All mice were housed under controlled temperature with a 12 hour light and 12 hour dark cycle. The mice were given standard animal feed (Altromin 1324, Germany) and clean water *ad libitum.*

### Animal euthanasia

**Figure 1:**
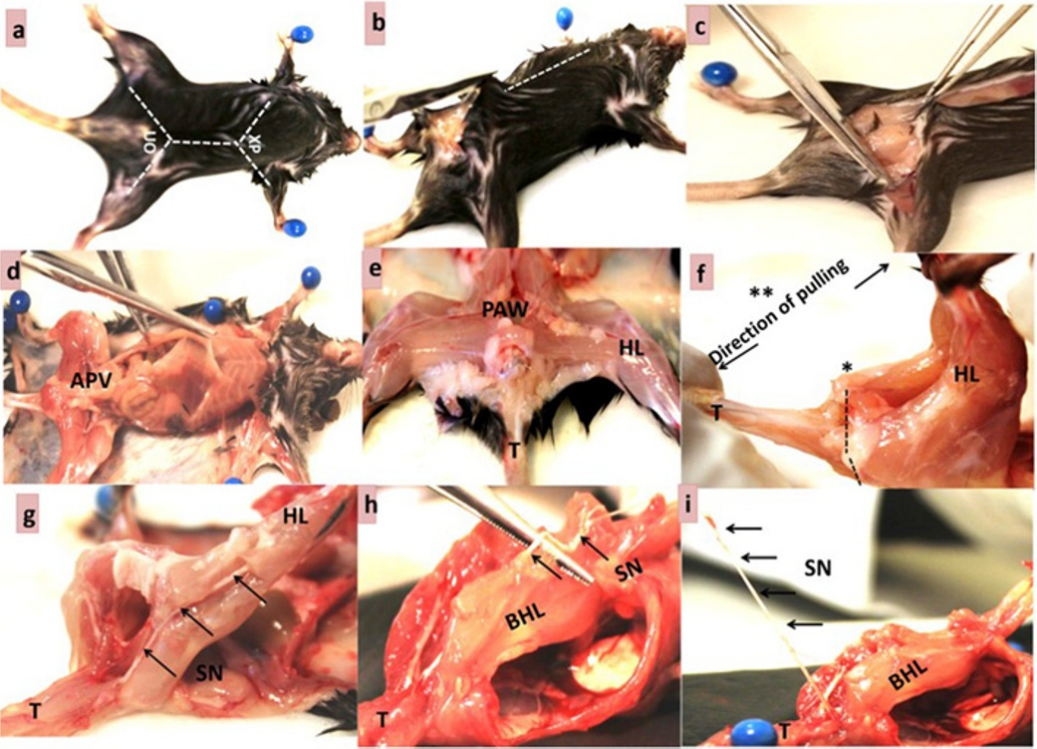
**Step-by-step approach for harvesting the sciatic nerve.** Mouse preparation and euthanasia **(a)**. Exposure of hind limb muscles and posterior abdominal wall **(b-e)**. Exposure of the sciatic nerve by pulling method **(f, g)**. Tracing and harvesting the sciatic nerve **(h, i)**. APV = Abdomino-pelvic viscera; BHL = Bent hind limb; HL = Hind limb; PAW = Posterior abdominal wall; SN = Sciatic nerve; T = Tail; UO = Urethral opening; XP = Xiphoid process; White dotted line = incision lines; Black dotted lines = small cut.

- The mice were deeply anesthetized with inhaled 4% (v/v) isoflurane prior to cervical dislocation to eliminate perception of pain.
- Each mouse was then pinned to a dissecting board in a supine position (Figure 1a). The skin was disinfected with 70% ethanol.

### Exposure of the posterior abdominal wall

This step is necessary for tracing the course of the nerve and to avoid obstruction by abdominal viscera.

- The ventral skin at the urethral opening was lifted using forceps and a small cut was made. A midline incision (*dotted white lines,* Figure 1a, b) was made from the urethral opening to the level of the sternal xiphoid process using surgical scissors (Figure 1b). A lateral incision was made from the urethral opening toward the knee on each side (Figure 1c).
- The ventral abdominal skin was pulled laterally, flipped and pinned to expose the peritoneum (Figure 1d). Similarly, the entire hind limb was skinned and the muscles (of the hind limb) were exposed (Figure 1d).
- The abdomino-pelvic viscera were removed using forceps and surgical scissors to expose the posterior abdominal wall (Figure 1d,e).

### Exposure and harvesting of the sciatic nerve

**Figure 2:**
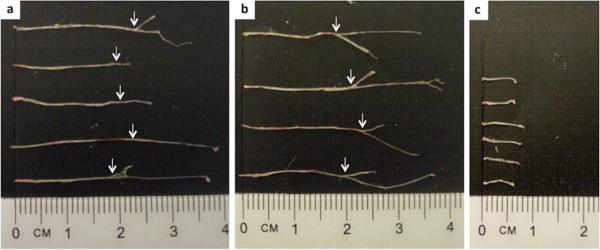
**Measurement of representative sciatic nerves harvested via the proposed method (a-b) and conventional mid-thigh incision approach (c)**. The point of bifurcation into tibial and common peroneal nerves is indicated by arrows **(a-b)**.

- Using a surgical blade, a deep cut was made at the base of the tail along the vertebral column (*dotted black lines*, Figure 1f) to detach the gluteal muscles.
- Both hind limbs were held together in position using the left hand and the base of the tail was held with the right hand (Figure 1f).
- The tail was gently pulled away from the hind limb at the point of the cut (Figure 1f) until the sciatic nerve was exposed (Figure 1g). The part of the nerve that runs along the lumbo-sacral region was exposed and appears as a thick whitish cord (arrows, Figure 1g). This step should be done with caution to avoid stretching the nerve. The course of the nerve was traced by splitting the hind limb muscles such as the gastrocnemius both distally and proximally (Figure 1h).
  [Caution: It is important to consider the amount of “pulling force” while pulling the tail away from the hind limbs as nerves are fragile and can be damaged by stretching or pressure. Excessive pulling force on the nerve could thin or break the nerve, as well as distort the histological morphology of the myelin.]
- A maximum length of the sciatic nerve was harvested (*lifted with forceps,* Figure 1i) from a point close to its origin in the sacral area through the popliteal fossa where it divides into two branches (Figure 2). In some instances, the tibial nerve, a larger branch of the sciatic nerve, was simply traced distally and harvested (Figure 2).
- The harvested nerve was then placed on a clean dark mounting board and gently straightened (Figure 2a,b).
- The nerve length was measured using a ruler and the reading was recorded (Figure 2a,b).

### Mid-thigh incision approach

- The mid-thigh incision method is well-documented [20] and the most commonly used conventional approach for harvesting the sciatic nerve from mice. A brief description of the mid-thigh incision method is given below (images of step-by-step dissection method are not shown).
- A small (~5.0 mm) vertical incision was made along the thigh using scissors and the skin was retracted laterally.
- The muscles of the posterior thigh (including the hamstring muscles) were split to expose the sciatic nerve, which appears as a thick whitish cord.
- The muscles were further split until the entire length of the sciatic nerve in the thigh region was exposed.
- The nerve was gently lifted using forceps and removed by cutting at the proximal and distal ends. The lengths of all harvested nerves were measured and recorded (Figure 2c).

## Findings

- Appearance and morphology: The harvested sciatic nerves were intact and appeared as thick whitish cords due to presence of the myelin sheath.
- Nerve length: The average lengths of the nerve obtained by the proposed method and the mid-thigh incision method were 22.60 ± 1.62 mm and 7.0 ± 0.76 mm, respectively. On average, the length of the nerve harvested using the proposed method was three times longer than the average length of the nerve obtained via the mid-thigh incision approach (Figure 2).

## Discussion

In many biomedical science studies, sufficient sciatic nerve tissue might be required for two or more different independent analyses. As such, the ability to harvest the maximum amount of the sciatic nerve should be considered. Most studies on the sciatic nerve showed isolation and harvesting of the nerve from the lateral aspect of the animal’s thigh [16, 18]. However, the length of the nerve obtained using this approach might be insufficient to provide tissue for different analyses and would in turn increase the number of experimental animals required for a given study. As such, alternative approaches that allow isolation of longer sections of sciatic nerve are needed. Here we describe an easy step-by-step approach that will yield maximal lengths of sciatic nerve from mice. Using this method, average lengths of sciatic nerve measuring ~22.6 mm could be obtained. A single sciatic nerve harvested using the proposed method would allow sufficient tissue for more than two downstream investigations, including light microscopy studies (paraffin-embedded tissue), immunohistochemistry studies (optimal cutting temperature-embedded tissue section), electron microscopy studies (resin-embedded tissue), RT-qPCR (RNA extraction) and western blotting analyses (protein extraction).

The comparison of the proposed method and the mid-thigh incision method clearly showed the differences in length of the harvested nerves, with the lengths obtained using the proposed method being significantly longer than that of the mid-thigh incision approach. The proposed harvesting method enables exposure of the lumbosacral region of the sciatic nerve, which is estimated to be longer than 10 mm.

Furthermore, mid-thigh dissection requires dissection of the right and left sides at different times, which lengthens the overall time needed to expose the nerve. In view of this, simultaneous exposure of both right and left sciatic nerves as described in this approach would reduce the time needed to harvest sciatic nerve tissue from mice. A technician who is well-acquainted with this dissection approach could harvest nerves from both sides in the shortest period of time. Moreover, while some knowledge of mouse anatomy is required for successful dissection of the sciatic nerve by either method, in the mid-thigh incision approach anatomical landmarks are used to dissect the thigh in order to expose the sciatic nerve and this could be challenging for researchers who are not well versed in veterinary anatomy. The proposed method requires minimal knowledge of detailed anatomy to perform, largely because both nerves can be exposed by pulling away the tail from the hind limbs.

## Conclusion

The dissection protocol described here provides an easy and time-saving approach for harvesting maximal lengths of sciatic nerve and requires no extensive dissection skills. In addition, this approach allows simultaneous exposure of both right and left sciatic nerves. The length of sciatic nerve obtained is sufficient to provide tissue for multiple analyses, which thus reduces the amount of resources and number of animals needed for these experiments.
